# Risk Prison and Hepatitis B Virus Infection among Inmates with History of Drug Injection in Isfahan, Iran

**DOI:** 10.1155/2013/735761

**Published:** 2013-04-22

**Authors:** Daneshmand Dana, Nokhodian Zary, Adibi Peyman, Ataei Behrooz

**Affiliations:** ^1^Infectious Diseases and Tropical Medicine Research Center, Isfahan University of Medical Sciences, Isfahan, Iran; ^2^Department of Gastroenterology, Integrative Functional Gastroenterology Research Center, Isfahan University of Medical Sciences, Isfahan, Iran; ^3^Nosocomial Infection Research Center, Isfahan University of Medical Sciences, Isfahan, Iran

## Abstract

*Objectives*. Hepatitis B virus (HBV) is a health problem among injection drug users (IDUs) in prison. The aim of this study is to evaluate the association of factors of incarceration with HBV infection in prisoners with history of drug injection in Isfahan, Iran. *Methods*. In a cross-sectional study, all IDUs inmates were enrolled. Sociodemographic characteristics and associated risk factors were obtained. Blood samples were collected and serological markers for HBV were analyzed. For data analysis, odds ratio and logistic regression were used. *Results*. Of the IDUs inmates, 970 subjects participated in the study. History of imprisonment (OR: 1.82, 95% CI: 1.28–2.57), multiple incarceration (OR: 1.43, 95% CI: 1.01–2.02), and total duration of imprisonment (OR: 2.70, 95% CI: 1.94–3.74) were significantly associated with prevalence of HBV among IDUs inmates. Multivariate analysis of associated factors showed that only total duration of incarceration is significantly associated with HBV infection. *Conclusion*. In conclusion, according to our results, multiple and duration of incarcerations will be considered as important risk factors of HBV infection in IDUs inmates. This fact makes it important to set some screening and prevention programs in prisons to decrease the risk of being infected and prevent the transmission of these diseases.

## 1. Introduction

Despite the availability of effective vaccination, hepatitis B virus (HBV) is still a major public health problem because of high chronicity rate and progression to cirrhosis and hepatocellular carcinoma [1]. Two million people have been infected with HBV and about 350 million people are estimated to be carriers worldwide [2]. The HBV will transmit via different ways such as mother to child transmission, blood and HBV contaminated injection equipments, intravenous drug use (IDU), and venereal transmission [3, 4]. Intravenous drug users (IDUs) are at high risk for infection with several blood-borne pathogens such as HBV, hepatitis C virus (HCV), and human immunodeficiency virus (HIV). IDUs often have coinfections of at least two of these diseases [5–7]. Prevalence of chronic hepatitis B infection in IDUs is determined about 3.2% [8]. In a study done by Tavakkoli et al. results showed that the most important risk factors of HBV infections in IDUs are imprisonment, male sex, and past history of bisexual relationship [9]. In the other hand, HBV infection and other blood-borne diseases among prison inmates are higher than the general population and maybe it is because of the over representation of IDUs and high-risk addiction-related behaviors, having multiple sexual partners and homosexuality, prison life style and limited educational opportunities of this population group [10–12]. During the last few years prevalence of blood-borne diseases such as HBV among prisoners have been investigated. Different studies show that the overall HBV infection ranges from 1.8% to 62% among adult inmates [12–14]. Prevalence of hepatitis B surface antigen (HBsAg), antihepatitis B core antibody (HBcAb), and antihepatitis B surface antibody (HBsAb) in the incarcerated women in Isfahan were determined 1.2%, 7.4%, and 12.9%, respectively [15]. Regarding to these facts, studies and intervention programs for special population groups such as prisoners especially those with history of intravenous drug using are needed to evaluate their infection risk and set up proper prevention and control strategies. Few studies on this population have been conducted in Isfahan, Iran, and considering the limited data on the risk factors of HBV among IDUs prison inmates, the aim of this study is to evaluate the association of frequency and duration of incarceration with HBV infection in prisoners with history of drug injection in Isfahan, Iran.

## 2. Methods

A cross-sectional study was conducted in 2 prisons of Isfahan. All the prisoners with history of drug injection (one time or more drug injections) in those centers were entered into the study on March 2009. The data were obtained from interview and blood testing. 

The prisoners were advised that participation in the study is on a voluntary basis, and an informed consent was obtained. The prisoners were assured that the results of their blood tests will never be reported to any organizations or person. After the explanation of the purpose of the study, all the prisoners were invited to participate. Participants answered a questionnaire with demographic characteristics such as age, marital status, education, and history of traveling to another country. The data about risk factors such as history of being incarcerated before the present time, frequency and duration of incarcerations were obtained from prisoners documents in the prison. Validity of the questionnaire was evaluated by specialists, and its reliability was confirmed by Cronbach alpha = 0.74. 

Blood samples were collected from each participant by health personnel of the prison setting. They were trained on safety blood sampling and filling questionnaire. Blood samples were then sent to the laboratory of Infectious Diseases Research Center during 3 hours in cold box to be tested to detect the following serological markers for HBV infection: HBsAg, HBcAb, and HBsAb. These procedures were performed by enzyme-linked immunosorbent assays (ELISA, Dia.Pro, Italy).

### 2.1. Data Analysis

Statistical analysis was performed using SPSS for windows (Version 16.0, 2007, SPSS Inc, Chicago, IL, USA). The Students *t*-test (for continuous variable) and Chi square test (for categorical variables) were used to compare variables and to evaluate associations between HBV positivity and associated factors. 

These data were estimated by the odds ratio in univariate analysis and also multiple logistic regression to estimate adjusted odds ratio (AOR) and 95% confidence intervals (CI). Statistical significance was assessed at the 0.05 probability level in all analyses. All the values are given as mean ± standard error (mean ± SE) or numbers (%). 

### 2.2. Ethics

The research protocol was approved by the Ethical committee of Isfahan University of Medical Sciences in Iran.

## 3. Results

A total of 970 IDUs male prisoners participated in the study. The mean ± SD of drug using duration for the participants was 12.52 ± 7.41 years. The mean ± SD age of the participants was 36.60 ± 0.31 years (median: 35, range: 22–71). Of the participants, 521 (55.4%) were married, and 143 (15.2%) had history of traveling to another country.  Table 1 is summarizing the sociodemographic characteristics of all the participants.

Of the 970 participants, 264 (27.2%) were positive for any of HBV serological markers, and 706 (72.8%) individuals were serologically negative for all HBV markers and are classified as susceptible group to HBV infection. Frequency of seropositive individuals for HBsAg, HBsAb, and HBcAb were 32 (3.3%), 188 (19.4%), and 135 (13.9%), respectively. 

Of the participants, 671 (71.5%) had the history of being incarcerated before the present time. Mean frequency of being incarcerated (multiple incarcerations) and total duration of incarceration for the study sample were 4.43 ± 0.17 times and 5.05 ± 0.17 years, respectively. The mean multiple and total duration of incarcerations in HBsAg positive subjects were 5.46 ± 0.91 and 5.04 ± 0.80, respectively and were not statically significantly different from the means of HBsAg negative prisoners (4.39 ± 0.18 and 5.05 ± 0.17, resp., *P* > 0.05). Mean multiple and total duration of incarcerations were significantly different between HBcAb positive (5.70 ± 0.59 times and 6.30 ± 0.49 years, resp.) and negative (4.18 ± 0.17 times and 4.81 ± 0.17 years, resp.) subjects (*P* = 0.002 and 0.001, resp.). 

The mean multiple and total duration of incarcerations in HBsAb positive subjects were 5.67 ± 0.53, and 7.39 ± 0.47, respectively, and were statically significantly different from the means of HBsAb negative prisoners (4.11 ± 0.17 and 4.47 ± 0.17 resp., *P* < 0.05). Comparison of risk factors between the subjects who are positive for any serological markers and negative prisoners is reported in Table 2.

## 4. Discussion

This study was conducted to evaluate the association of duration and frequency of being incarcerated with the prevalence of hepatitis B virus (HBV) infection among IDUs prison inmates in Isfahan. The results showed that the longer duration of being incarcerated is associated with higher prevalence of HBV infection. Also previous history of being incarcerated and higher frequency of incarcerations are associated with higher prevalence of HBV infection. There is no similar study in Isfahan, but in a study conducted by Nokhodian et al. [15] on the female prisoners in Isfahan, results showed that there was no association between number of arrests and HBV markers seropositivity. In their study, the frequency of HBsAb seropositivity among prisoners with higher number of being arrested was more than the others but not statically significantly different. Their study sample was 163, and it can be a big limitation for their study. Also their study was only conducted on females. Another study showed the similar results and revealed no association between having prison history and HBV infection among females with illegal social behavior in Isfahan [16]. There is a limited data on the association of HBV infection and incarceration in the IDUs in Iran, but as we know HBV is a blood-borne disease like HCV and HIV with the same ways of transmission. In a study, results showed that incarceration is a major risk factor for blood-borne infection among intravenous drug users [17]. Another study by Zamani et al. [18] showed that risk of HCV infection transmission as a blood-borne disease is related to the length of lifetime incarcerations (OR, 3.44; 95% CI, 1.68–7.06).

Studies from other parts of the world showed similar results. In a study in Brazil by Stief et al. [12], results showed that frequency of being incarcerated more than three times is associated with 1.9-fold risk of HBV infection. Another study in Berlin showed that positive history of imprisonment is associated with 1.5-fold risk of HBV seropositivity, and syringes sharing in prison more than 50 times increases the risk by 3.9-fold. Their results showed that history of syringes sharing in prison is significantly associated with HBV, HCV, and HIV infections. They mentioned that prisons are places where IDUs who continue to inject are at a high risk of developing blood-borne disease [19]. Another study in Nigeria showed that both duration of being in prison and history of previous incarceration are significantly associated with HBV seropositivity [20]. The results of our study were similar to most of the previous surveys and showed that multiple and long time duration of incarcerations are risk factors for HBV infections in intravenous drug users inside prisons and it needs special attentions. 

## 5. Conclusion

In conclusion, according to our results, multiple and duration of incarcerations will be considered as an important risk factors of being infected with blood-borne diseases such as HBV infection in prison inmates with history of drug injection. Injection drug use during incarceration is common and carries a substantial risk of infection with blood-borne viruses such as HBV. Prevention strategies are needed to reduce the risk of infection among incarcerated injecting drug users. Also it is important to set some screening and prevention programs especially primary preventions in prisons to decrease the risk of being infected and prevent the transmission of the disease.

## Figures and Tables

**Table 1:** Socio-demographic characteristics of the participants.

Characteristics	Number (%)
Marital status	
Single	419 (44.6%)
Married	521 (55.4%)
Education level	
Illiterate	23 (3.7%)
Elementary school	236 (37.8%)
Junior high school	253 (40.4%)
High school	31 (4.9%)
Diploma	68 (10.9%)
University	15 (2.3%)
History of traveling	
Yes	143 (15.2%)
No	795 (84.8%)

**Table 2:** Hepatitis B virus infection associated risk factors among prisoners.

Factors	Positive	Negative	OR (95% CI)	*P*	AOR (95% CI)
History of imprisonment					
Yes	201 (29.9%)	470 (70.1%)	1.82 (1.28–2.57)	0.001	0.32 (0.06–1.66)
No	51 (19.0%)	217 (81.0%)			
Multiple incarceration^†^ (time)					
	5.43 ± 0.41	3.99 ± 0.18	—	0.000	—
<4.5	125 (27.9%)	323 (72.1%)	1.43 (1.01–2.02)	0.042	1.17 (0.80–1.71)
≥4.5	77 (35.6%)	139 (64.4%)			
Total duration of imprisonment^†^ (year)					
	6.64 ± 0.36	4.41 ± 0.18	—	0.000	—
<5	99 (20.9%)	374 (79.1%)	2.70 (1.94–3.74)	0.000	2.47* (1.72–3.54)
≥5	113 (41.7%)	158 (58.3%)			

Data are given as number (%) or mean ± SE, 95% CI: 95% confidence interval, OR: odds ratio, AOR: adjusted odds ratio, ^†^categorization is based on mean of total study sample, *P > 0.05.
